# Functional vision promotes quality of life and socio-emotional functioning in visually impaired children and adolescents

**DOI:** 10.1371/journal.pone.0342443

**Published:** 2026-08-04

**Authors:** Guido Catalano, Federica Morelli, Serena Grumi, Daria Paini, Francesco Decortes, Ilaria Scognamillo, Maria Eleonora Reffo, Roberta Zumiani, Sandra Strazzer, Elena Cocchi, Livio Provenzi, Sabrina Signorini, The VIDA Group

**Affiliations:** 1 Department of Brain and Behavioural Sciences, University of Pavia, Pavia, Italy; 2 Developmental Neuro-Ophthalmology Unit, IRCCS Mondino Foundation, Pavia, Italy; 3 Department of Psychiatry, Autism Spectrum Disorders and Related Conditions Service, Lausanne University Hospital (CHUV), Lausanne, Switzerland; 4 Developmental Psychobiology Lab, IRCCS Mondino Foundation, Pavia, Italy; 5 Robert Hollman Foundation “Consultation and Support for the Development of Visually Impaired Children”, Padova, Italy; 6 Abilnova Cooperativa Sociale, Trento, Italy; 7 Scientific Institute, IRCCS E. Medea, Centro Regionale per l’ipovisione in Età Evolutiva, Bosisio Parini, Lecco, Italy; 8 Fondazione David Chiossone per Ciechi ed ipovedenti ONLUS, Genova, Italy; No institution, UNITED KINGDOM OF GREAT BRITAIN AND NORTHERN IRELAND

## Abstract

Vision plays a pivotal role in development. Congenital and early acquired visual impairment (VI) may adversely impact several domains related to everyday functioning, such as social and academic inclusion, autonomies and quality of life. In recent years, the inclusion of these dimensions as key targets of the assessment and re-habilitation of paediatric VI has received increasing consensus. This cross-sectional study enrolled patients attending 5 centres specialized in paediatric VI care and aimed to draw an integrated profile of functioning, autonomies, quality of life and emotional-behavioural domains in a large cohort of children with VI. 91 caregivers of as many visually impaired children (age range 6–18, mean age 11.05 years, 47 females) – split into two age groups: 6–10 years; 11–18 years – were enrolled in an on-line survey including three validated questionnaires to assess children’s: a) autonomies, *via* a Patient Reported Outcome Measure (PROM) tool; b) socio-emotional and behavioural problems; c) quality of life (QoL). Clinical and socio-demographic information were also collected. Both age groups showed medium-high average level of autonomy in every investigated area as well as appropriate socio-emotional, behavioral, and QoL scores. Interestingly, adolescents showed lower scores in autonomies compared to the younger age group overall. Autonomy score significantly associated with better QoL and less socio-emotional and behavioural problems, both internalizing and externalizing. Our results highlight the importance of assessing autonomy in the context of paediatric VI re-habilitation as they largely associate and potentially impact on young patients’ psychological well-being.

## Introduction

Vision is the dominant sensory modality in early development, supporting the acquisition of motor, cognitive, and social-emotional skills [1,2]. As such, the burden of a congenital or early acquired visual impairment (VI) may extend beyond visual functions per se (i.e., oculomotor, perceptual and visuo-cognitive aspects [3]) and affect a child’s short and long-term developmental trajectory [4]. In fact, early visual deprivation can lead to a delay in the acquisition of developmental milestones (e.g., walking, talking, regulating one’s emotions) compared to sighted children. If not adequately addressed, this developmental gap may tend to progressively widen over time, resulting in reduced functional vision (i.e., the everyday life functioning of a person with residual vision) [5,6], social participation and, ultimately, perceived quality of life (QoL) [7].

Of the many areas of a person’s development and functioning that can be hindered by congenital or early acquired VI, socio-emotional and behavioural outcomes warrant special attention, since vision is crucial for accessing social cues and sustaining early interpersonal exchanges. Indeed, visually impaired people tend to present higher rates of psychological difficulties, though the underlying mechanisms are still debated. For example, the lack of experience of eye contact, access to facial expressions and imitation might lead to socio-emotional and communicative deficits since early childhood [8,9]. Consequently, as these children progress through school and adolescence, they may experience growing challenges in integrating with peers and, later in life, face barriers to participation and inclusion in educational and employment contexts. Focus groups with children and parents developing vision-specific quality-of-life measures have highlighted psychosocial and school-related difficulties as key concerns [10], while epidemiological studies have reported lower employment rates with increasing levels of visual disability [11]. Moreover, external factors—such as educational opportunities, workplace accessibility, and parental support—can either facilitate or hinder participation and adjustment [12].However, these studies do not provide information on the long-term outcome of VI, leaving a gap in our understanding of the early mechanisms and effects of visual deprivation on adaptation and mental health. Most studies rely on small sample sizes or anecdotal evidence and often focus on specific eye conditions. Furthermore, reports often include people affected by non-isolated peripheral VI [13,14], introducing confounding factors, such as neurological conditions, that make it difficult to identify a consistent socio-emotional and behavioural profile of this population. Moreover, the absence of specific outcome measures has hampered efforts to clarify the contribution of visual function and/or functional vision to quality of life and socio-emotional adjustment. A recent work by Semrov and colleagues [15] pointed out how better socioeconomic status, attending mainstream school, the absence of other chronic conditions, and better functional vision are predictors of better quality of life (QoL) perception among visually impaired children. However, remains unclear how visual function and functional vision interact in shaping children’s adaptation. Such information could be useful to better define whether rehabilitation programs should prioritize sensory function, functional skills or an integrated approach.

The recent development and validation of the VIDA (Visual Impairment Developmental Autonomy) scale, a standardized measure designed to assess functional vision (specifically, developmental autonomies, which define the level of functional independence of an individual) in VI pediatric populations, offered an opportunity to address this gap.

Building on this tool, the present study aimed to: i) describe the socio-emotional and behavioural profile of a large cohort of children with congenital or early-acquired VI; and ii) inquire whether aspect of visual function (namely, visual acuity, which measures the degree of visual impairment) and/or functional vision (namely, functional independence) are associated with better perceived quality of life and socio-emotional and behavioural outcomes. Specifically, we hypothesize that both visual function and functional vision would contribute to more favorable socio-emotional and behavioural outcomes, as well as to better perceived quality of life.

## Materials and methods

Data were collected between February and September 2023 at the Developmental Neuro-Ophthalmology Unit of IRCCS Mondino Foundation, Pavia, Italy. The present study was part of a wider project aimed at developing and validating the VIDA scale [16].

The study was approved by the local Ethical Committee of Pavia, (Fondazione IRCCS Policlinico San Matteo, Pavia, Italy) on 5th June 2020, with Protocol Number p-20200048762. All the procedures followed the Declaration of Helsinki ethical principles for research involving human subjects.

Visually impaired children and adolescents were enrolled with their parents from five institutions in northern Italy dedicated to the care and re-habilitation of VI: Developmental Neuro-ophthalmology Unit of the IRCCS Mondino Foundation (Pavia, Italy), Fondazione David Chiossone (Genoa, Italy), IRCCS Eugenio Medea (Bosisio Parini, Italy), Fondazione Robert Hollman (Padova, Italy), AbilNova Cooperativa Sociale (Trento, Italy). Families were approached during clinical visits and subsequently contacted by phone to invite voluntary participation. Parents received an email containing an invitation link to the software Qualtrics XM. Reading and flagging an informed consent was required for participation. A code was associated with each participant after the software access to allow anonymization. Parents were asked to fill in a) a form with socio-demographic information, b) the VIDA scale (see Grumi et al, 2022 [17]; and Morelli et al, 2025 [16]) c) the TNO-AZL Children Quality of Life (TACQoOL) [18], d) Child Behaviour Checklist (CBCL 6–18) [19]. Both theTACQoL and CBCL 6–18 had been previously validated and standardized for the Italian pediatric population. Thus, their Italian versions was chosen for this study as they are widely used in clinical research. was required to fill the questionnaires.

### Participants

Inclusion criteria were: child age between 6 and 18 years, child and parent full mastery of Italian language, peripheral visual impairment (i.e., involving pre-geniculate structures and pathways) resulting in low vision. Vision was assessed, defined, and categorised throughout a multidisciplinary assessment protocol [3] according to the Italian legislative system (G.U. Serie Generale, n. 93 del 21 Aprile 2001). This implied that visual acuity was tested with standardised methods (symbolic or literal optotype, chosen based on the age) at a 3m distance and categorised as mild (0.7–0.5 logMAR), moderate (0.7−1 logMAR), severe (1–1.30 logMAR) low vision or blindness (>1.30 logMAR). Exclusion criteria included a diagnosis of developmental delay/intellectual disability, based on standardised evaluations chosen according to the age and degree of VI (Griffiths Scales of Child Development, Reynell-Zinkin Scales, Wechsler Intelligence Scales). Further exclusion criteria were the presence of neuromotor disorder, central nervous system involvement, or chronic comorbidities.

### Measures

#### Functional vision level (VIDA scale).

The VIDA scale is a questionnaire developed to measure functional vision, assessing the developmental autonomies (or functional independence) level of visually impaired children and adolescents [17]. It has recently been validated in an Italian pediatric population, showing good internal consistency (Cronbach’s α > .80) and adequate convergent validity with similar questionnaires [16]. For each item of the VIDA scale, participants were asked to attribute a score of 0 (the subject cannot perform the task), 1 (the subject can perform the task with help) or 2 (the subject can perform the task without help). Mean sub-scores for each area (*table manners, clothing, personal hygiene, orientation and mobility, and socio-affectivity*) and a total mean score were computed for each age version of the instrument (i.e., 3–5; 6–10; 11–18 years). According to VIDA scale age ranges, the sample has been divided in two groups: 43 patients aged 6–10 years old, 48 patients aged 11–18 years old.

#### Quality of life.

On the basis of the child’s age, participants completed the Children’s Quality of Life questionnaire (TACQOL [18,20]). The 56-item TACQOL covers seven domains of health-related quality of life: physical complaints, motor functioning, autonomous functioning, cognitive functioning, social functioning, positive moods, and negative moods. It was not possible to administer the more specific Vision-Related Quality of Life Instrument for Children and Young People with Visual Impairment [21] as it was not available in our Country at the time of the study.

#### Child socio-emotional and behavioural problems.

The Child Behaviour Checklist (CBCL 6–18) is a 113 items and extensively used questionnaire to assess children’s (aging 6–18 years old) adjustment. The respondent (usually a parent or another caregiver) is asked to estimate the degree to which his or her child exhibits a set of problem behaviours using a 3-point Likert scale ranging from 0 (not true) to 2 (very or often true). It provides scores for internalizing (e.g., withdrawn-depressed) and externalizing problems (e.g., aggressive behaviour) and a total score on the child’s overall psychological adjustment.

### Statistical plan

Descriptive statistics (i.e., frequencies, means, and standard deviations) were used to describe demographics and clinical variables as well as score distributions of the variables of interest. Comparisons between the two age groups were performed using the Wilcoxon-Mann-Whitney test for ordinal variables or the *t*-test for continuous ones. For continuous variables, data normality was assessed using the Shapiro–Wilk test. The equality of variances was evaluated using Levene’s test, and when this assumption was violated, the Welch correction was applied. To test the influence of visual function (i.e., visual acuity), a set of analyses of variance (ANOVAs) were performed to test differences between sub-groups with different visual impairment (i.e., blind, severe or moderate/mild low vision – see Participants section for the visual impairment classification) on quality of life and emotional-behavioural issues variables. Pearson’s bivariate correlations coefficients were used to test the association between functional vision (i.e.,VIDA scale score), quality of life, and emotional-behavioural issues. Statistical analyses were performed using IBM SPSS version 25.

## Results

### Sample characteristics

Of the 139 eligible families approached, 91 (participation rate = 65.46%) agreed to participate. Parents of a total of 91 of patients with peripheral VI, aged 6–18 years old (mean age 11.05 + /- 3.69 years) participated in the study signing all the questionnaires. Perceptual characteristics of the participants were so represented: 19 were blind, 19 had severe low vision, 30 moderate low vision, 23 had mild low vision. The two age groups did not differ significatively in gender composition (f% 48.8 in 6–10 years old cohort, f% 54.2 in 11–18 years old cohort; χ² = 1.284; p = .526) and visual acuity levels (χ² = 1.623; p = .654). Etiology distribution is reported also in Table 1, the most common cause of visual impairment was Inherited Retinal Distrophy and ocular malformations. All the enrolled patients received comprehensive visual re-habilitation and had access to visual aids and community support (e.g., support teacher, sports associations) provided by a complement of multidisciplinary professionals working together, centred around the needs of the child and family. Therefore, it was not possible to assess the effect of re-habilitation towards the three considered domains (i.e., autonomies, quality of life, socio-emotional and behavioural problems). None of the socio-demographic variables significantly associated with any of the three domains investigated.

**Table 1:** Sample characteristics.

Variable	Total sample (n = 91) f (%) or M (sd; range)	Age 6–10 (n = 43) f (%) or M (sd; range)	Age 11–18 (n = 48) f (%) or M (sd; range)
Sex	47 (51.6) females	21 (48.8)	26 (54.2)
Age	11.05 (3.69; 6-18)	7.81 (1.45; 6-10)	13.96 (2.44; 11-18)
Diagnosis	IRD = 30 (33)	IRD = 17 (39.5)	IRD = 13 (27.1)
	ROP = 14 (13.4)	ROP = 6 (14)	ROP = 8 (16.7)
	MAC = 11 (12.1)	MAC = 2 (4.7)	MAC = 9 (18.8)
	OCA = 8 (8.8)	OCA = 4 (9.3)	OCA = 4 (8.3)
	ONH = 8 (8.8)	ONH = 6 (14)	ONH = 2 (4.2)
	INS = 5 (5.5)	INS = 3 (7)	INS = 2 (4.2)
	CC = 5 (5.5)	CC = 2 (4.7)	CC = 3 (6.3)
	CG = 3 (3.3)	CG = 1 (2.3)	CG = 2 (4.2)
	MMA = 3 (3.3)	MMA = 1 (2.3)	MMA = 2 (4.2)
	RB = 2 (2.2)	RB = 1 (2.3)	RB = 1 (2.1)
Visual Acuity	Blind = 19 (20.9)	Blind = 7 (16.3)	Blind = 12 (25)
	Severe low vision = 19 (20.9)	Severe low vision = 10 (23.3)	Severe low vision = 9 (18.8)
	Moderate low vision = 30 (33)	Moderate low vision = 16 (37.2)	Moderate low vision = 14 (29.2)
	Mild low vision = 23 (25.3)	Mild low vision = 10 (23.3)	Mild low vision = 13 (27.1)
Ongoing re-habilitation	76 (83.5)	43 (100)	33 (68.8)
Re-habilitation specific for autonomies	63 (69.2)	32 (74.4)	31 (64.6)
Mothers’ education (years)	13.82 (3.63; 5-22)	14.79 (3.02; 8-22)	12.96 (3.93; 2-22)
Fathers’ education (years)	12.55 (3.26; 8-18)	12.56 (2.82; 8-18)	12.54 (3.64; 8-18)
Mothers’ occupation status	72 (79.1) working	33 (76.7) working	39 (81.3) working
Fathers’ occupation status	88 (96.7) working	43 (100) working	45 (93.8) working

Note: F: frequency. M: mean. IRD: inherited retinal dystrophy. ROP: retinopathy of prematurity. MAC: microphthalmia. anophthalmia. coloboma spectrum. OCA: oculo-cutaneous albinism. ONH: optic nerve hypoplasia. INS: infantile nystagmus syndrome. CC: congenital cataract. CG: congenital glaucoma. MMA: methylmalonic acidemia. RB: retinoblastoma.

### Developmental autonomies, socio-emotional and behavioural profiles

**Fig 1:**
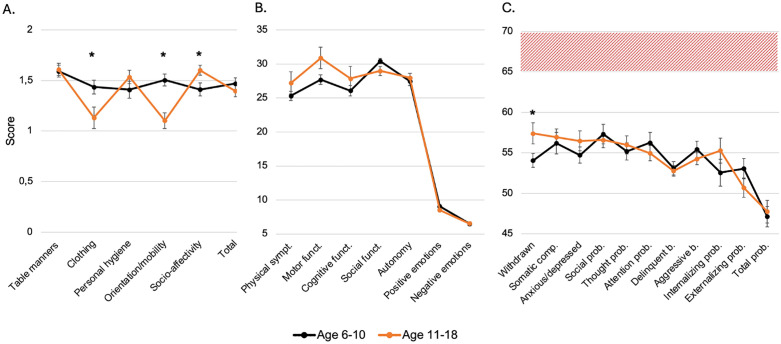
Cohort profiling across a) autonomy level, b) quality of life perception c) emotional-behavioural problems. A) VIDA scale domains: Table Manners, Clothing, Hygiene, Orientation and Mobility, Socio Affectivity, total VIDA score. **Interpretation**: Levels of autonomy for each area are represented in terms of mean values, considering the specific items for each age range. Note: the mean scores range from 0 “the subject cannot perform the tasks” to 2 “he/she can perform the tasks wihout help”. B) TACQoL scales: physical complaints, motor functioning, cognitive functioning, social functioning, autonomy, positive moods, negative moods **Interpretation**: the sum scores may range from 0 to 32 for physical symptoms, motor functioning, cognitive functioning, social functioning, and autonomous functioning. For Positive and Negative emotions areas the scores vary between 0 and 16 cutoffs. C) CBCL subscales: Withdrawn, Somatic symptoms, Anxiety, Social Problems, Thought problems, Attention problems, Rule Breaking Behaviour, Aggressive Behaviour, Internalizing Problems, Externalizing problems, Total Problems **Interpretation**: T-scores ≥ 64 are considered in the clinical range for the broadband scales, T–scores ≥ 70 are considered in the clinical range for the syndrome scales. ‘Borderline’ elevations range from T scores 60–63 and 65–69 on the broadband and syndrome scales, respectively.

The VIDA scale scores exhibited on average good autonomy levels among the two age ranges (age group 6−10 mean VIDA total score = 1.470; age group 11−18 mean VIDA total score = 1.396). However, adolescents performed significantly worse than children in *Clothing* (*t*(79.9)= 2.4, *p* = .019, Cohen’s d = 0.54) and *Orientation and mobility* (*t*(85.2)= 4.08, *p*= < .001, Cohen’s d = 0.88); while showed a better functioning for the socio-affectivity sub-scale (*t*(89)= −2.36, *p* = .02, Cohen’s d = −0.50). Perceived QoL levels were similar *t*o published normative data for the scale in all the investigated areas. Note that for TACQOL scoring, specifically for positive and negative mood areas, the scores vary between 0−16 cutoffs. Moreover, no significant differences between the two age groups are reported (see Fig 1B). Emotional behavioural profiling did not show clinical mean scores in any of the investigated CBCL domains (see Fig 1C) and no significant differences emerged between 6−10 and 11−18 age groups, except for the Withdrawn/Depressed domain (*t*(80)= −2.02, *p* = .046, Cohen’s d = −0.45).

### Association between visual function (visual acuity), emotional-behavioural problems, and perceived quality of life

No significant differences emerged between children with different acuity for quality of life as well as for socio-emotional and behavioural problems, except for the group 11–18 years group, where these statistical significant differences emerged: children with severe VI had lower scores than counterparts with moderate/mild VI on CBCL socio-emotional scale *somatic complaints* (*F(2)*= 3.39; *p* = .043, η^2^ = .079) and for *externalizing problems* (*F(2)*=3.28; *p* = .047, η^2^ = .077). No significant differences emerged for the quality of life.

### Association between Autonomy levels (functional vision), emotional-behavioural problems, and perceived quality of life

Statistically significant correlations emerged between autonomy levels, socio-emotional and behavioural problems, and quality of life scores (Tables 2 and 3).

**Table 2:** Pearsons’ correlations between VIDA and CBCL for both age groups.

	Age 6–10						Age 11–18					
	a)	b)	c)	d)	e)	f)	a)	b)	c)	d)	e)	f)
1)	−0,233	−0,132	−0,163	−0,316	−,410	−0,284	0,042	−0,239	−,386*	−,433*	−,442*	−,360
2)	−,507*	−,349	−,364	−,604*	−,350	−,495*	0,072	0,118	−0,187	−0,114	−0,071	−0,028
3)	−0,189	−0,082	−0,249	−0,288	−0,303	−0,257	−0,015	−0,106	−,348	−0,206	−,332	−0,234
4)	−,688*	−,608*	−,597*	−,713*	−,533*	−,722*	−0,036	−0,071	−0,239	−0,125	−,328	−0,178
5)	−,644*	−,504*	−,503*	−,623*	−,571*	−,651*	−0,039	−0,084	−0,215	−0,053	−0,224	−0,14
6)	−,687*	−,540*	−,554*	−,604*	−,514*	−,666*	−0,11	−0,116	−,356	−0,267	−,312	−0,273
7)	−0,034	−0,004	−0,074	−0,104	−0,293	−0,117	0,037	0,035	−0,118	−0,01	0,1	0,009
8)	−,407*	−0,241	−,334	−,426	−,406	−,415	0,142	−0,011	−0,216	−0,07	−0,106	−0,06
**9)**	−,345	−0,192	−0,328	−,477*	−,356	−,389	0,032	−0,001	−0,272	−0,197	−0,266	−0,156
**10)**	−,391	−0,256	−0,321	−,422	−0,321	−,392	0,157	0,045	−0,139	0,015	−0,055	0,012
**11)**	−,520*	−,409	−,478*	−,567*	−,437*	−,555*	0,103	0,043	−0,187	−0,094	−0,171	−0,061

Note. *significat correlations after the application of Bonferroni corrections. VIDA scale domains: a) Table Manners, b) Clothing, c) Hygiene, d) Orientation and Mobility, e) Socio Affectivity, f) total VIDA score. CBCL subscales: 1) Withdrawn, 2) Somatic symptoms, 3) Anxiety, 4) Social Problems, 5) Thought problems, 6) Attention problems, 7) Rule Breaking Behaviour, 8) Aggressive Behaviour, 9) Internalizing Problems, 10) Externalizing problems, 11) Total Problems.

**Table 3:** Pearsons’ correlations between VIDA and TACQOL scales quality of life for both age groups.

	Age 6–10						Age 11–18					
	a)	b)	c)	d)	e)	f)	a)	b)	c)	d)	e)	f)
**12)**	0,208	0,198	,071	0,118	0,191	0,179	−0,04	0,047	−0,034	−0,105	−0,087	−0,042
**13)**	0,386	0,443*	0,345	0,295	0,325	0,421*	0,011	0,006	0,177	0,183	0,09	0,104
**14)**	0,508*	0,327	0,460*	0,432*	0,2	0,452*	0,035	0,02	0,015	−0,011	0,002	0,001
**15)**	0,265	0,15	0,162	0,307	0,269	0,262	0,02	0,157	0,154	0,358	0,423*	0,268
**16)**	0,105	0,029	−,046	,029	0,100	0,043	0,157	0,010	0,004	0,135	0,092	0,092
**17)**	0,339	0,398	0,469*	0,367	0,23	0,427*	0,156	0,278	0,185	0,385*	0,224	0,320
**18)**	−0,103	−0,064	−0,093	−0,03	−0,015	−0,073	0,121	−0,048	0,041	−0,149	−0,126	−0,045

Note. *significat correlations after the application of Bonferroni corrections. VIDA scale domains: a) Table Manners, b) Clothing, c) Hygiene, d) Orientation and Mobility, e) Socio Affectivity, f) total VIDA score. TACQoL scales: 12) physical complaints, 13) motor functioning, 14) cognitive functioning, 15) social functioning, 16) autonomous functioning 17) positive moods, 18) negative moods

## Discussion

This study stems from the validation process of the VIDA scale. Its primary objective was to assess the perceived autonomy, quality of life, and emotional-behavioural profiles of a large cohort of children and adolescents with peripheral visual impairment. Additionally, we sought to explore how QoL and emotional-behavioural issues are influenced by two factors: visual function (specifically the visual acuity level, which measures the degree of visual impairment) and functional vision (everyday life autonomies, which represent the functional independence).

### Developmental autonomies, socio-emotional and behavioural profiles

Overall, the autonomy level (assessed with the VIDA scale) in our cohort aligned with what is expected, and all domains of QoL and emotional-behavioural profiles presented non-clinical (i.e., non-pathological) average scores compared to available normative data.

However, adolescents exhibited significantly more withdrawal/depressive symptoms as well as lower autonomy scores in domains such as *clothing* and *orientation and mobility*. In addition, correlations between daily living autonomies (which contribute to functional independence), QoL and emotional-behavioural scores appeared weaker in this population/age range. Several considerations may be proposed based on these data, although definite conclusions cannot be drawn as it is the first study using these tools and it refers to a specific and relatively small sample. First, as children grow older, the demands on the visual system increase (e.g., they may need to process information faster, to read smaller print, to learn to drive, etc). Visually impaired children and adolescents may thus experience a reduced sense of independence, disclosing a potential discrepancy between visual function and functional vision: even a child with stable visual acuity or mild low vision may struggle with daily life activities that they’re challenged with. We may also hypothesize that the inability to accomplish these more complex tasks can result in poorer socio-relational skills and mental outcomes. Previous studies actually report an increased risk of depression and anxiety among visually impaired adolescents, emphasizing the complex interaction between sensory deprivation, functional disability, quality of life and emotional wellbeing [22,23].

Both empirical and theoretical interpretations could be given for the good level of autonomies, perceived quality of life and emotional behavioral indexes in our cohort. First, all participants in our cohort have been carrying out specific re-habilitation programs since early childhood at specialized centres for visual impairment. Such re-habilitation likely played a protective role on functional vision, mental well-being and QoL: besides specific trainings for autonomy, these children have always relied to a network of professionals taking care of their needs, intercepting their signs of distress, and working together with families and schools to adapt the environment to their vision level. Theoretically, our results are consistent with the “disability paradox” [24]. Such a construct expects that children with chronic diseases may perceive good psychosocial conditions independently from the severity of their impairment. This phenomenon has been demonstrated as linked to socio economic, familiar, and clinical factors [25].

A limitation of the present study is that outcomes were assessed exclusively through proxy reports, without including adolescents’ self-reports. Previous research has shown only moderate agreement between parent and self-reports, particularly for subjective dimensions of quality of life, which may not be fully observable by caregivers, and the level of accordance is debated [26–28]. Therefore, the findings should be interpreted with caution, as proxy reports may not completely capture adolescents’ self-perceived emotional well-being. Future studies adopting multi-informant approaches through accessible tools are warranted to better clarify these discrepancies, strengthen the validity of findings, and ultimately allow direct participation of patients in their care.

### Association between Autonomy levels (functional vision), emotional-behavioural problems, and perceived quality of life

Among the clinical factors that could have a role in enhancing a good perceived QoL and emotional-behavioural status, we investigated *functional vision. Functional vision* seems having a greater influence than *visual function.* While the degree of VI is – as expected [29] –associated with the capacity to acquire autonomies, it does not seem to relate with quality of life and emotional well-being. In other words, functional independence may buffer the potential negative impact of visual disability on QoL, emotions and behaviors.

Our findings contribute to an ongoing debate in the literature regarding the roles of visual function and functional vision in shaping quality of life. Leske et al. [30], using the PedEyeQ, reported a significant association between visual acuity and both functional vision and vision related QoL. In contrast, Semrov et al. [15], and Robertson et al. [21] found that functional vision and broader contextual factors, rather than the degree of visual acuity, were more strongly associated to perceived QoL. Our study extends this line of evidence by clarifying the role of daily living autonomies as a specific component of functional vision, highlighting their potential protective effect on emotional–behavioural outcomes and QoL in rehabilitative settings.

### Clinical and re-habilitative implications

This study discloses an association between functional independence and well-being in visually impaired children. These findings emphasize the importance of early autonomy training to support independence, resilience, and overall functioning. Enhancing functional vision may also mitigate the risk of poor emotional behavioural outcomes. As children feel more self-efficient, they may develop greater self-esteem and self-control, increasing their ability to cope more effectively with their impairment [22].

**Fig 2:**
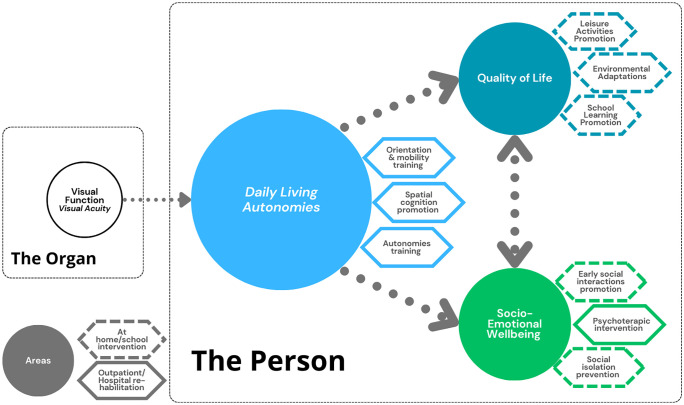
Visual function, functional vision, quality of life and emotional-behavioural aspects relation. Proposal of a multidisciplinary and multidimensional re-habilitation model for visually impaired children. The angled boxes report the re-habilitation interventions for each area and represent the different dimensions (outpatient/hospital, school, home) in which these functions shall be promoted.

However, this alone may not be sufficient. A multidisciplinary rehabilitation approach, as proposed by the ICF-CY guidelines on comprehensive patient care, could be critical in supporting the well-being of visually impaired children and their families. The care model for VI patients depicted in Fig 2 (based on the one firstly described by Morelli et al. [31]) proposes a re-habilitation path that considers the child’s developmental profile and guided by continuous collaboration between professionals and key figures in the child’s life (e.g., parents, teachers) [32–34].

This study may be one of the first steps towards more precise guidelines for the care of these patients. Identifying strengths and challenges in children and adolescents with VI is crucial to prioritize potential targets of intervention and personalise their care. Future studies are needed to further explore this topic, including adolescents’ self-reports on QoL and mental wellbeing, control groups, and based on a prospective design to draw specific developmental trajectories also in relation to the type and the duration of the rehabilitative intervention.
